# *Ab initio* molecular dynamics simulations of liquid water using high quality meta-GGA functionals†
†Electronic supplementary information (ESI) available. See DOI: 10.1039/c6sc04711d


**DOI:** 10.1039/c6sc04711d

**Published:** 2017-02-27

**Authors:** Luis Ruiz Pestana, Narbe Mardirossian, Martin Head-Gordon, Teresa Head-Gordon

**Affiliations:** a Chemical Sciences Division , Lawrence Berkeley National Laboratory , Berkeley , USA . Email: thg@berkeley.edu; b Kenneth S. Pitzer Center for Theoretical Chemistry , Department of Chemistry , University of California , Berkeley , USA; c Departments of Chemistry , Bioengineering , Chemical and Biomolecular Engineering , University of California , Berkeley , USA

## Abstract

We have used *ab initio* molecular dynamics (AIMD) to characterize water properties using two meta-generalized gradient approximation (meta-GGA) functionals, M06-L-D3 and B97M-rV, and compared their performance against a standard GGA corrected for dispersion, revPBE-D3, at ambient conditions (298 K, and 1 g cm^–3^ or 1 atm). Simulations of the equilibrium density, radial distribution functions, self-diffusivity, the infrared spectrum, liquid dipole moments, and characterizations of the hydrogen bond network show that all three functionals have overcome the problem of the early AIMD simulations that erroneously found ambient water to be highly structured, but they differ substantially among themselves in agreement with experiment on this range of water properties. We show directly using water cluster data up through the pentamer that revPBE-D3 benefits from a cancellation of its intrinsic functional error by running classical trajectories, whereas the meta-GGA functionals are demonstrably more accurate and would require the simulation of nuclear quantum effects to realize better agreement with all cluster and condensed phase properties.

## Abstract 2

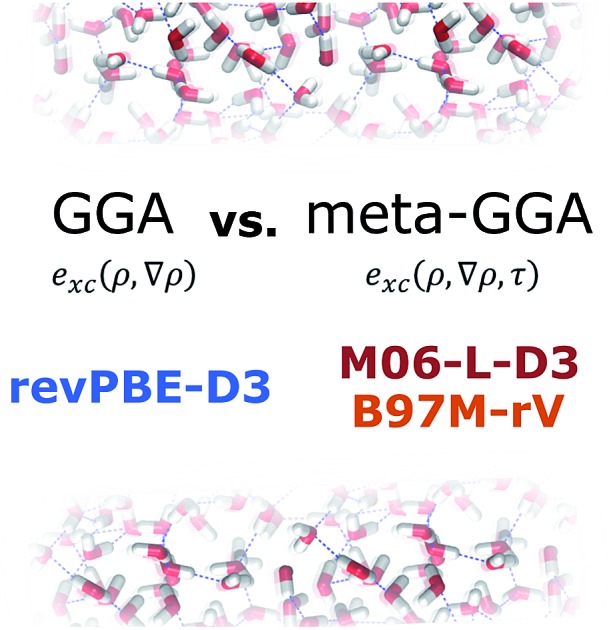
We have used *ab initio* molecular dynamics (AIMD) to characterize water properties using two meta-generalized gradient approximation (meta-GGA) functionals, M06-L-D3 and B97M-rV, and compared their performance against a standard GGA corrected for dispersion, revPBE-D3, at ambient conditions (298 K, and 1 g cm^–3^ or 1 atm).

## Introduction

The anomalous properties of bulk water, *e.g.* temperature of maximum density and divergence of thermodynamic response functions upon cooling at low temperature,^1^ emerge from the collective behavior of associated water molecules.^2^,^3^ And while it is well known that bulk water near ambient conditions is a thermally distorted tetrahedral liquid,^4^ under more asymmetric environments such as nanoconfinement or near solutes and interfaces, the properties of water remain a matter of debate.^5^–^9^

Atomistic simulation approaches, such as molecular dynamics (MD) with empirical force fields, have already provided fundamental insight into the structure, dynamics, and thermodynamics of water.^10^–^13^ For bulk water, empirical force fields continue to improve through introduction of better physics such as polarization,^14^–^17^ the evolution away from hand-tuning to better global optimization of parameters using expanded training data sets^18^,^19^ or rapidly converging many-body expansions,^20^ and a philosophy of optimizing these parameters for a few select properties such as density and heats of vaporization over a range of state points^21^–^23^ in order to capture the changes in the hydrogen-bonded network that impact other properties, such as transport or solvation. This approach applied to water is showing that the resulting force fields are capable of describing the spectroscopic properties,^24^,^25^ the correct ordering of the ice phases and its melting point,^22^ the isothermal compressibility or diffusion constant over a range of temperatures,^19^,^23^ as well as more accurate solution properties such as the structural organization of peptides in water^26^ or solvation free energies.^27^,^28^ An important point to emphasize is that these conclusions can only be drawn when derived from convergence of statistical properties through sufficient sampling, and as empirical force fields become more complex, they benefit from new algorithms and software that increase sampling statistics.^29^–^31^ However, although both computationally efficient and surprisingly accurate, most empirical force fields are unable to describe the electronic reorganization (*e.g.* bond breaking) in a chemical reaction.

*Ab initio* molecular dynamics (AIMD),^32^–^34^ where the potential energy surface (PES) is calculated every step from first principles electronic structure methods, allows the simulation of aqueous chemical reactions in arbitrarily complex environments. Despite the real success^35^–^37^ of extensive efforts that started more than two decades ago,^38^,^39^ the AIMD field is still working towards a model chemistry and simulation protocol that performs as well as an empirical force field for describing just bulk liquid water. Kohn–Sham density functional theory (DFT) provides reasonable physical accuracy at a moderate computational cost, thus it is the most widely employed electronic structure method in AIMD simulations.^35^–^37^ DFT-based AIMD is typically carried out at the generalized-gradient approximation (GGA) level, as it offers an excellent compromise between the crude physical accuracy of the local density approximation (LDA) and the heavy computational cost of hybrid functionals that incorporate a fraction of exact exchange. Early on, popular GGA functionals such as Becke–Lee–Yang–Parr (BLYP)^40^,^41^ or Perdew–Burke–Ernzerhof (PBE)^42^ were shown to give reasonable geometries for water molecules,^43^ but captured poorly the relative energies of water clusters,^44^ which in the condensed phase lead to a low density and over-structured liquid with glassy dynamics at ambient conditions.^45^–^51^ This poor performance has been rationalized in the light of the intrinsic limitations of GGA functionals^52^—namely, the so-called delocalization error (DE)^53^ and the lack of non-local correlations necessary to describe dispersion interactions. The DE leads to systematic errors in the monomer deformability^54^ (*i.e.* red shifts in intramolecular vibrational frequencies) and thus overestimation of the dissociation energies of dimers extracted from the condensed phase where distortions are substantial.^55^,^56^ By promoting excessive delocalization of the proton, the DE also contributes to the strengthening of the hydrogen bonds in liquid water.

Despite their relatively weak nature, van der Waals (vdW) forces are critical to the behavior of water in the condensed phase,^57^ making the lack of non-local correlation in density functionals an important shortcoming. Fortunately, several practical approaches have been proposed to circumvent this problem.^58^,^59^ Popular dispersion corrections such as Grimme's DFT-D pair-wise semiempirical corrections,^60^,^61^ or the non-local correlation functionals VV10 62,63 (and its cousin rVV10 64), vdW-DF,^65^ or the Tkatchenko and Scheffler vdW,^66^,^67^,^78^ are computationally cheap and generally improve the description of liquid water by GGAs.^47^–^50^,^68^–^71^ Dispersion interactions offer stabilizing forces with no angular dependence, which tend to increase the population of water molecules between the first and second coordination shells^68^ thereby counteracting the effect of the DE. Although dispersion corrections constitute in most cases a step in the right direction (*e.g.* increase of bulk density, less overstructure of the radial distribution function, reduction in the caging that slows down the diffusivity of liquid water, *etc.*), the effect depends on the particular functional and the specific correction employed.^68^ For example, while adding vdW-DF corrections to BLYP results in a general improvement in the condensed phase, when added to PBE, the second coordination shell becomes significantly disrupted, and the overall description of the liquid is further worsened.^48^,^69^ A particularly telling story of success is the GGA functional revPBE,^72^ which performs quite poorly by itself but when used with D3 dispersion corrections describes liquid water remarkably well,^49^ at least when used with classical molecular dynamics.

Besides the improvement granted by dispersion corrections, adding a fraction of exact exchange in the so-called hybrid functionals can mitigate the DE in semi-local functionals. For example, the hybrid functional PBE0 73) is able to reproduce the monomer deformation energies calculated from highly accurate CCSD(T) calculations^55^-, and the vibrational spectrum of PBE0 water is in much better agreement with experiments than the spectrum calculated using semi-local functionals.^74^ Furthermore, because the one-body contribution is the leading term in the error of the many-body expansion, the binding energies are also significantly improved upon addition of exact exchange.^56^ Although hybrids offer some improvements over semi-local functionals for liquid water, the equilibrium density under ambient conditions is still too low, and neither the RDFs nor the diffusivity are in quantitative agreement with experiments.^75^,^76^ This is in part because hybrids also lack non-local correlations.^77^ DiStasio *et al.* showed that if pairwise TS-vdW dispersion corrections^79^ are added to PBE0, and the simulation is run at 330 K (justified as a crude way to reproduce nuclear quantum effects), at least the structural properties are in good agreement with experimental results.^79^ Despite the steady growth of computational power and the recent development of efficient algorithms to compute the exact exchange^80^— factors that have enabled the use of hybrid functionals in AIMD simulations^51^,^74^–^76^,^79^—hybrids are still considerably more expensive than semi-local functionals. Furthermore, because in general hybrids require larger basis sets to perform adequately due to a more severe case of basis set superposition error (BSSE) relative to semi-local functionals,^81^ the cost is further exacerbated.

In this work, we focus on meta-GGA density functionals, where the kinetic energy density is used in addition to the density and its gradient, such that higher accuracy is expected while mostly maintaining the computational advantages of semi-local GGA approximations. In extensive benchmarking across multiple properties including dimer binding energies, barrier heights, and thermochemistry, the good performance of the meta-GGAs M06-L^82^ with Grimme's D3 dispersion correction^61^ and the newly developed B97M-rV^83^,^84^ that competes directly in accuracy with earlier hybrid functionals, stand out such that we have used them to investigate the AIMD description of bulk ambient water.

Here, we have calculated a number of ambient water properties: the equilibrium density, the radial distribution functions, self-diffusivity, the infrared (IR) spectrum, liquid dipole moments, and characterizations of the hydrogen bond network. We find that all three functionals have overcome the problem of the early PBE and BLYP functionals that erroneously found AIMD-DFT ambient water to be highly structured, but they differ substantially among themselves in agreement with experiment on this range of water properties. While on the face of it AIMD using revPBE-D3 reproduces the properties of liquid water with surprising accuracy, we show directly using water cluster data up through the pentamer that this is due to fortuitous cancellation of its fairly large intrinsic error with the error in performing simulations using classical trajectories, *i.e.* lack of nuclear quantum effects (NQE). By contrast, the meta-GGAs are inherently more accurate, and in fact require the inclusion of NQE to bring them into line with the experimental cluster and liquid state properties, whereas this additional physics will worsen the agreement with experiment for revPBE-D3. This work evaluates for the first time the performance of some of the best semi-local meta-GGA functionals currently available, filling in a crucial gap in AIMD studies of liquid water, and awaits the inclusion of NQEs to expose the inherent weaknesses or full strengths of different DFT functionals for future condensed phase simulations.

## Computational details

For the electronic structure calculations, we adopt the Gaussian plane-wave (GPW)^85^ approach to DFT^86^ implemented in the subroutine QUICKSTEP^87^ of the freely available program CP2K.^88^ We use the standard implementation in the library LIBXC^89^ for the exchange and correlation of the functionals revPBE,^72^ M06-L,^82^ and B97M-rV.^83^,^84^ We use Grimme's DFT-D3 dispersion corrections with zero-damping^61^ for revPBE and M06-L implemented in CP2K. Because all the D3 corrections are with zero-damping, we refer to them as just D3, instead of the alternative notation D3(0). For the non-local correlation functional of B97M-rV, we use the rVV10 implementation included in CP2K with parameters *b* = 6.0 and *C* = 0.01, the same parameters used in the original implementation of the functional with VV10.^84^ These parameter values have been recently recommended also for rVV10 based on validation across the very large dataset.^84^ It was also shown that at the complete basis set limit B97M-rV in fact slightly exceeds the parent B97M-V functional.^84^ For the GPW calculations in CP2K we use the Goedecker–Teter–Hutter (GTH) pseudopotentials (PP) to represent the core electrons.^90^,^91^ We use the same PP for all the functionals, which was originally optimized for the GGA functional PBE (PBE-PP). We use a family of Gaussian basis sets optimized for DFT calculations in molecular systems, MOLOPT,^92^ which are also specifically designed for use with GTH-PPs. The basis sets in the MOLOPT family contain primitive diffuse functions that are critical for the accurate description of hydrogen bonds and electronegative atoms, and is implemented with a multi-grid system with 5 levels, which gives optimal performance for the MOLOPT basis sets.

We validate the GPW implementation of the functionals by comparing them to all-electron calculations done with the Q-Chem software package.^93^ We show that the errors in the binding energies of the S22 94,95 set, which contains 8 dispersion-bound, 7 hydrogen-bonded, and 7 mixed non-covalent interactions, are in sufficient agreement (∼0.1*k*_B_*T* error) with the near basis set limit all-electron reference calculations (Tables S1 and S2†). Furthermore, we show in Table S3† that the error incurred in the relative energies of isomerization in the WATER20 96 dataset (using updated reference values^97^) by using the generic PBE-PP (instead of a functional-optimized PP) is negligible, which justifies in practice the use of the same PBE-PP for all the functionals. Further details regarding these calculations are given in the in the ESI.†

Obtaining accurate and reliable results from a GPW calculation also requires convergence of the real-space integration grid, whose finesse is determined by the energy cutoff.^87^ A cutoff value of 280 Ry has been a standard value in AIMD simulations of water in the condensed phase using isochoric ensembles (NVT or NVE),^98^–^100^ although recently, higher cutoffs ranging from 400 to 800 Ry have been also suggested.^101^,^102^ In the isobaric-isothermal ensemble (NPT), a larger cutoff is often required due to grid sensitivity issues (*e.g.* discontinuities in the number of grid points) when the volume of the simulation cell can fluctuate.^45^,^103^ Although values as large as 1200 Ry have been proposed,^45^,^103^ a more recent study suggests that the density and structural properties converge for 600 Ry.^47^ Here we use an energy cutoff of 400 Ry for AIMD simulations in the NVT and NVE ensembles, and 800 Ry in simulations where the size of the simulation cell can fluctuate.

While the BSSE is reasonably well understood in water clusters and other hydrogen-bonded complexes,^104^–^106^ how it affects the condensed phase dynamics and configurational landscape remains unclear. For example, while some AIMD studies have suggested insensitivity to basis set size beyond a double-zeta basis set,^99^ or even slightly worse results on going from a double-zeta to a triple-zeta basis set^48^ due to some fortuitous cancellation of errors for the smaller basis, Lee and Tuckerman showed using a discrete variable representation basis set that a considerably better description of BLYP liquid water could be achieved in the complete basis set limit.^107^–^109^ Since small basis sets and moderate energy cutoffs are required in condensed phase simulations for computational efficiency, and since the meta-GGA functionals were originally developed and optimized using fine grids and large basis sets, we have also performed a number of tests on the S22 and WATER20 96 datasets to understand the errors introduced by running under our simulation protocol (Tables S4–S6†). The mean signed percentage errors (MSPE) of the binding energies for the S22 and WATER20 sets, shown in Tables S4 and S5† respectively, reflect a consistent trend expected from basis set superposition error (BSSE).^110^,^111^ In other words, any case that exhibits under-binding in the all-electron Q-Chem simulations, systematically improves in the CP2K calculation with smaller basis set, whereas cases that display over-binding get worse. A clear example of this is M06-L-D3, whose MSPE for the S22 dataset goes from –7.96% (Q-Chem) to 1.65% (CP2K, mTZV2P, 400 Ry).

For the S22 set (Table S4†), the root mean squared error (RMSE) of the binding energies for the S22 dataset (Table S4†) is fairly insensitive to the BSSE in general, displaying errors that are close to the Q-Chem benchmark calculations. Also, relative differences in errors between the different basis sets and energy cutoffs are small for the S22 dataset. In the case of WATER20 (Table S5†), while the RMSE for B97M-rV are comparable to the Q-Chem benchmark, (*e.g.* 1.64 kcal ml^–1^ (Q-Chem) *vs.* 2.8 kcal mol^–1^ (CP2K, mTZV2P, 400 Ry)), the RMSE decreases dramatically for revPBE-D3 (9.70 kcal mol^–1^ (Q-Chem) *vs.* 3.03 kcal mol^–1^ (CP2K, mTZV2P, 400 Ry)), and increases substantially for M06-L-D3 (4.15 kcal mol^–1^ (Q-Chem) *vs.* 11.58 kcal mol^–1^ (CP2K, mTZV2P, 800 Ry)). Interestingly, the large differences in RMSE between 400 Ry and 800 Ry observed for M06-L-D3 for both basis sets, may be indicative of the sensitivity of the Minnesota functional to the grid size.

Besides the binding energies that may be of limited relevance in the condensed phase, we also calculated the relative isomerization energies of the WATER20 dataset (Table S6†). For the relative energies, we observe, in general, very similar errors between the CP2K calculations and the Q-Chem benchmarks, although again some differences can be observed for M06-L-D3 between 400 Ry and 800 Ry. Overall, these results support that the structure and spectroscopic properties of the functionals are reasonably well represented by the GPW method for different basis sets and energy cutoffs.

The AIMD simulations performed in this paper are within the Born–Oppenheimer approximation, where the electronic structure of the system is solved using GPW at every step during the dynamics. Most studies of liquid water using the GPW approach employ the TZV2P basis set^87^ based on the results of VandeVondele *et al.*^99^ Here, we also use the mTZV2P basis set in the AIMD simulations. We use an energy convergence threshold of 10^–12^ and a convergence tolerance for the SCF cycle of 10^–6^ during the AIMD simulations, and 5 × 10^–7^ for the simulations on the benchmark data sets. For each functional, after an equilibration if ∼5 ps, we carry out a production run in the NVT ensemble for another 40 ps using the mTZV2P basis and a 400 Ry energy cutoff. For all the simulations, we use a cubic cell with 64 water molecules at density 1 g cm^–3^ (*L* = 12.42 Å). The temperature is maintained at 298 K by a massive Nosé–Hoover chain thermostat^112^,^113^ with a time constant of 3 ps.

To evaluate the dynamical properties and avoid possible artifacts introduced by the thermostat, starting from equilibrated configurations in the NVT ensemble, we also run for at least another 40 ps in the NVE ensemble. We investigate the equilibrium density of the different functionals using *ab initio* hybrid Monte Carlo (AI-HMC). The MC volume moves offer the advantage that the pressure is explicitly included in the acceptance rules, avoiding the large fluctuations and slow convergence of AIMD in the NpT ensemble using a virial. To avoid discontinuities in the energy that appear when grid points are added or removed upon a change in the simulation cell size,^45^ we constrain the grid density at a given energy cutoff in the AI-HMC simulations. We use the grid in a cubic box of size *L* = 12.42 Å (which corresponds to liquid water at 1 g cm^–3^) as the reference grid. All the simulations are run at 298 K and 1 atm. We use an energy cutoff of 800 Ry and the mTZV2P basis set. For the hybrid moves (*i.e.* short AIMD runs) within the AI-HMC simulations, we use the NVT ensemble and a time step of 0.5 fs. To reduce the number of *ab initio* energy evaluations, a biasing potential is used for generating trial moves. Specifically, we use the SPC/E^114^ empirical force field through the molecular mechanics subroutine FIST of CP2K. A total of 8 pre-sampling moves are attempted between each *ab initio* energy evaluation. The maximum displacements of the MC moves are 1000 steps for the AIMD runs, and 50 Å^3^ as maximum volume change. These values were chosen after some preliminary testing to achieve acceptance rates of approximately 50% for the different move types. The probabilities of attempting a hybrid move or a volume move are 70% and 30%, respectively, which gives a similar number of accepted hybrid and volume MC moves. To validate the AI-HMC simulations, we also calculated the equilibrium density of revPBE-D3 using AIMD simulations in the NPT ensemble for different basis sets and energy cutoffs (Fig. S1†). Further details of these calculations are offered in the ESI.†

We perform a vibrational analysis for small water clusters, from the dimer to the hexamer (including the cage, prism, book and ring isomers) using Q-Chem (Table S7†). For the eight water clusters, full geometry optimizations were carried out with each functional, followed by a harmonic frequency calculation. The def2-QZVPPD basis set was used for the frequency calculations, along with a very fine integration grid (250 radial shells with 974 angular grid points per shell) for local exchange–correlation functionals, and the SG-1 integration grid for the VV10 nonlocal correlation functional. We also calculate the bonded OH vibrational frequencies of the water clusters, from the dimer to the pentamer in CP2K using a 25 Å side periodic cubic box, the mTZV2P basis set, and a cutoff of 400 Ry.

In addition, we perform short classical AIMD simulations of the water clusters, from the dimer to the pentamer, to obtain their IR spectrum. All the simulations of the water clusters are performed in a cubic simulation box of side *L* = 9.857 Å, using the mTZV2P basis set, and a 400 Ry energy cutoff, and at a temperature of 298 K. For each water cluster we start from the optimized structure, and perform a short run of 5 ps in the NVT ensemble. After that, we restart the simulation in the NVE ensemble for another 5 ps, which is sufficient time to correctly sample the OH stretching modes, and use the trajectory to calculate the IR spectrum. The vibrational frequencies of the OH-stretching band are shown in Table S9.†

## Results

**Fig. 1:**
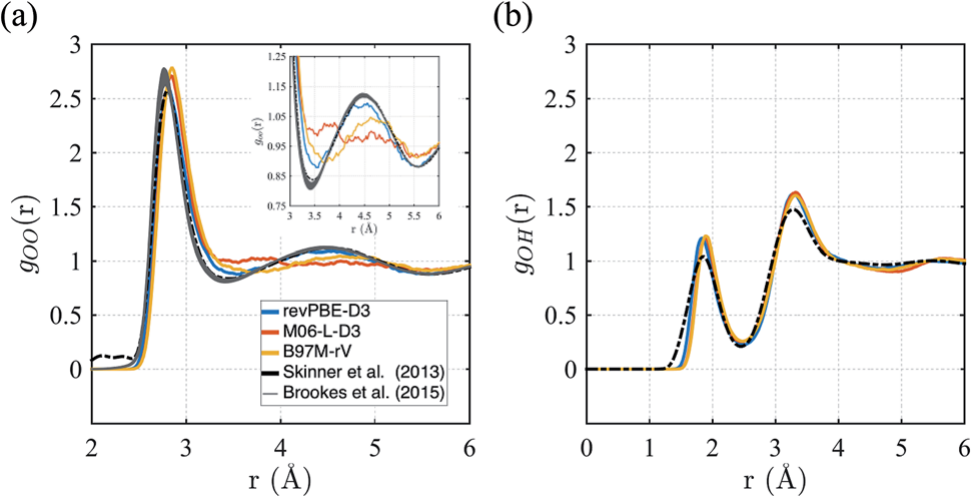
Radial distribution functions for revPBE-D3, M06-L-D3, and B97M-rV compared to recent experimental results.^116^,^117^ (a) *g*_OO_(*r*), the inset in panel (a) focuses on the region corresponding to the interstitial region and the 2^nd^ hydration shell. (b) *g*_OH_(*r*).

First we consider the straight performance of the three density functionals on simulating ambient water (1 g cm^–3^ and *T* = 298 K) with classical trajectories, the typical standard for most AIMD applications of bulk water. To characterize the structure, we have calculated their intermolecular oxygen–oxygen, *g*_OO_(*r*) and oxygen–hydrogen *g*_OH_(*r*) radial distribution functions (RDFs) and compared them to experimental data in Fig. 1. For *g*_OO_(*r*) we note that the original experiment by Skinner *et al.*^115^ has unphysical density in regions where correlations should be forbidden, giving rise to unphysical values of the isothermal compressibility. We therefore include the family of *g*_OO_(*r*)'s derived by Brookes and Head-Gordon^116^ that utilized two restraints on the allowable *g*_OO_(*r*) functions which must conform to both very low experimental errors in the intensity at high values of momentum transfer, *Q*, and the need to satisfy reasonable estimates of the isothermal compressibility at low-*Q*. For *g*_OH_(*r*), we compare against the RDF generated from neutron scattering and reported by Soper.^117^

The *g*_OO_(*r*) curves of all three functionals are quite good compared to earlier GGA functionals that were typically over-structured, and revPBE-D3 stands more accurate than the meta-GGAs (Fig. 1a). While B97M-rV is systematically shifted to larger *r* values and exhibits a shallower 1^st^ trough, the M06-L-D3 functional fails to reproduce the 2^nd^ peak altogether, indicating a complete loss of tetrahedral structure. Regarding the *g*_OH_(*r*), all three functionals exhibit very similar and slightly over-structuring behavior (Fig. 1b), although the locations of the peaks agree well with the experimental results.

**Fig. 2:**
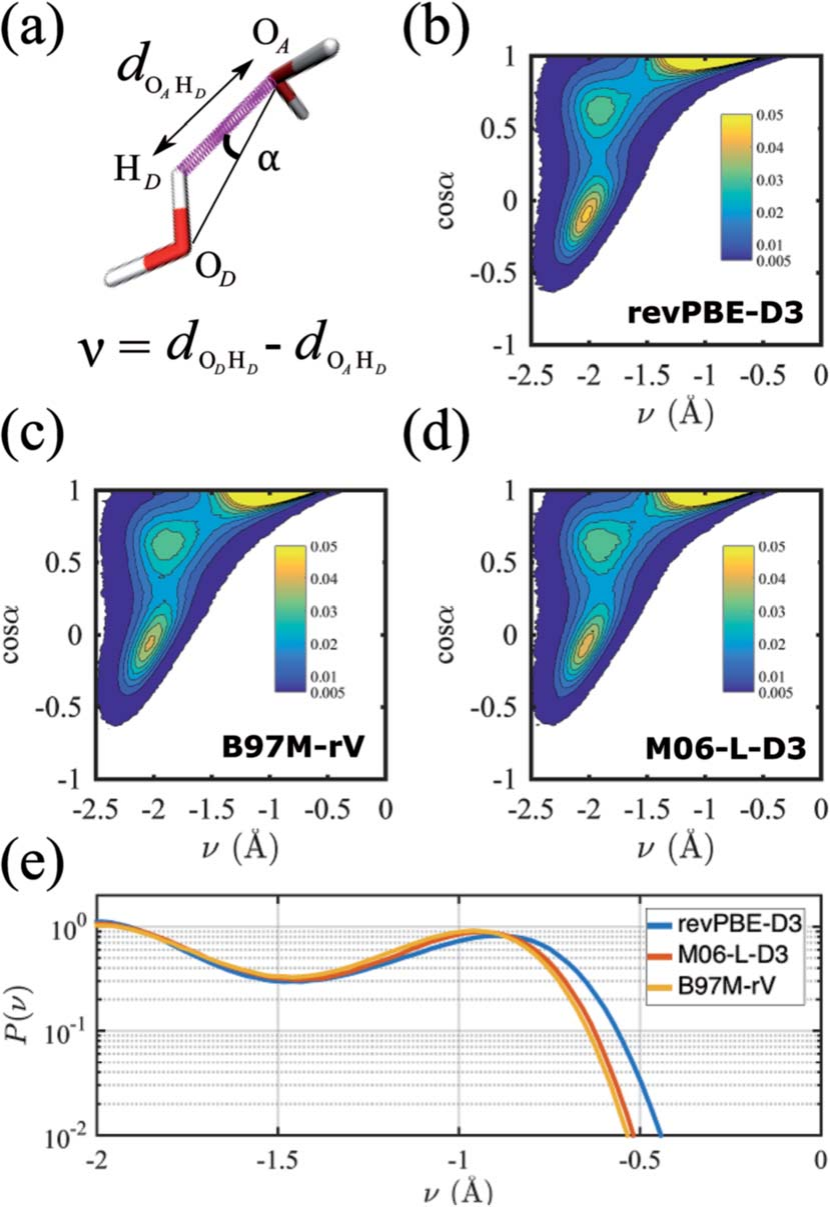
Analysis of the hydrogen bond network. (a) Schematic representation of the relevant variables: the O_D_–O_A_–H_D_ hydrogen bond angle *α*, and the proton transfer coordinate, *v* = *d*_O_D_H_D__ – *d*_O_A_H_D__. The color scale for the probability heat maps is also shown. Panels (b)–(d) are the joint probability distributions of *α* and *ν* for the different functionals. Panel (e) shows the log-probability distributions of just the proton transfer coordinate.

A critical aspect governing the properties of water is the hydrogen-bonded network,^118^ which is only indirectly captured in the radially averaged structure of the RDFs. To gain further insight into the properties of the hydrogen-bonded network of the three functionals, we analyze the probability distribution of the hydrogen bond and the proton transfer coordinate in Fig. 2. Although there are several ways to describe a hydrogen bond,^119^,^120^ here we use the angle *α*, defined by the donor oxygen O_D_, the acceptor oxygen O_A_ and the donor hydrogen H_D_, as well as the proton transfer coordinate *v* = *d*_O_D_H_D__ – *d*_O_A_H_D__, which captures the fluctuations of the proton along the hydrogen bond (Fig. 2a).

The resulting joint probability distributions *P*(*v*, cos *α*) for each of the three functionals are shown in Fig. 2(b)–(d), where the most probable region corresponds to ideal hydrogen bond values of the angle *α* ≈ 0 while the other two probable regions correspond to non-ideal or bent hydrogen-bonded configurations. The distributions are very similar for the three functionals, and are in good agreement with previous AIMD simulations without NQE.^121^ If NQE were included, we expect all would achieve non-zero probability of *ν* > 0, indicating the presence of transient autoprotolysis.^121^ When we integrate just over the proton transfer coordinate we find that the tail of the distribution is slightly fatter in the case of revPBE-D3, indicating that the proton fluctuations along the hydrogen bond direction are larger for the GGA functional (Fig. 2e). A higher delocalization of the proton will translate on average to a larger molecular dipole moment, which in turn would lead to stronger hydrogen bonds. This is corroborated with the average values of the molecular dipole moments calculated from the Wannier centers, which are 2.87 D, 2.72 D, and 2.79 D for revPBE-D3, B97M-rV, and M06-L-D3, respectively. This also supports the RDF profiles in which the *g*_OO_(*r*) is more structured for revPBE-D3 compared to the meta-GGAs.

**Fig. 3:**
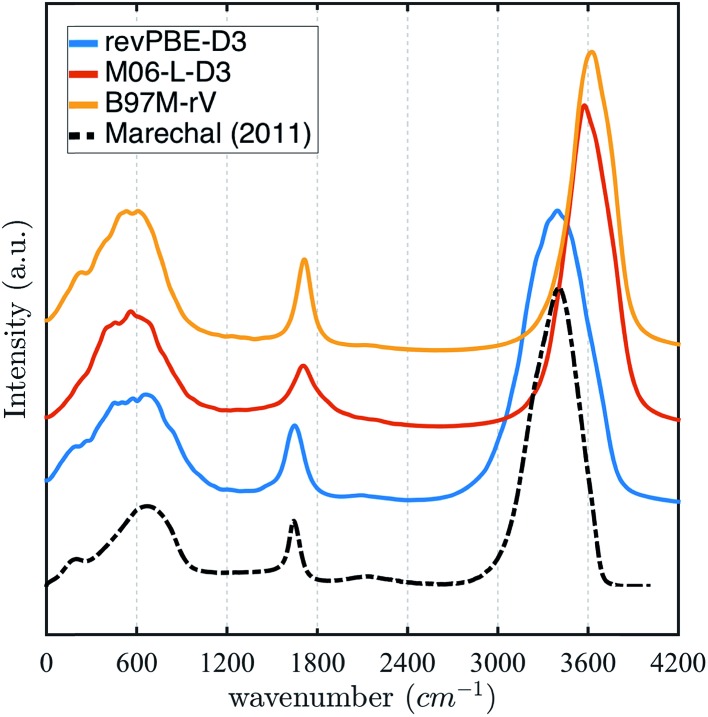
Infrared (IR) spectrum calculated for revPBE-D3, M06-L-D3, and B97M-rV compared to the experimental values.^122^ For visualization purposes, we have rescaled the experimental curve such that the intensity of the peak of the faster vibrational mode coincides with that of revPBE-D3.

The stronger hydrogen bonding exhibited by the revPBE-D3 functional compared to the meta-GGAs also plays out in the observed dynamical properties in the condensed phase, first evident in the IR spectrum of liquid water^122^ (Fig. 3). While we find that the peak for the water intramolecular modes in the liquid is very accurately captured by revPBE-D3, both meta-GGAs exhibit blue shifts of ∼200 cm^–1^ and ∼80 cm^–1^ for the stretching and bending mode, respectively. This is consistent with the fact that the intramolecular vibrational motions of the water molecule are stiffer for the meta-GGA functionals relative to revPBE-D3 (Table 1), which leads to weaker intermolecular hydrogen bonding in the water network evident from the RDFs and hydrogen-bonding probabilities.

**Table 1:** Vibrational frequencies of liquid water for revPBE-D3, M06-L-D3, and B97M-rV measured from the IR spectrum, and compared to experiment

IR mode	revPBE-D3	B97M-rV	M06-L-D3	Experiment
Bonded O–H	3405	3622	3577	3404.0
Angle bend	1648	1713	1707	1643.5
Libration (rocking)	667.8	570.1	560.3	686.3
Hydrogen bonding	∼231	221	—	∼200.0

All three functionals predict very similar low frequency librational modes, but in this case their peaks are shifted ∼80 cm^–1^ towards lower frequencies, indicating that the intermolecular hydrogen bonding network permits more permissive rocking motions. Although, both revPBE-D3 and B97M-rV exhibit the characteristic shoulder in the THz region—associated with the low frequency stretching and bending of intermolecular hydrogen bonds—M06-L-D3 lacks this feature, although a longer dedicated simulation might change this outcome.

**Fig. 4:**
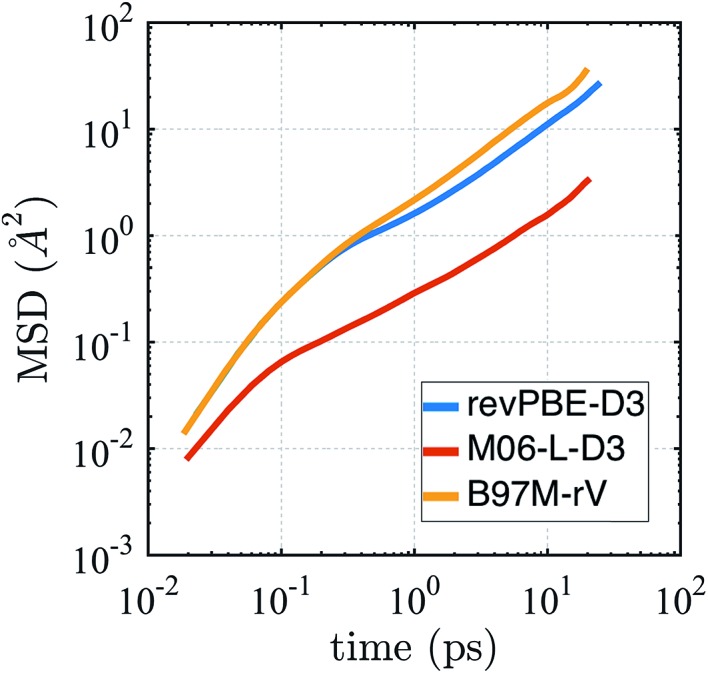
Mean squared displacement (MSD) from AIMD simulations in the NVE ensemble for revPBE-D3, M06-L-D3, and B97M-rV on a log–log scale.

The self-diffusion coefficient in Fig. 4 is calculated from the average slope of the mean square displacement (MSD) of the particles using the Einstein relation1
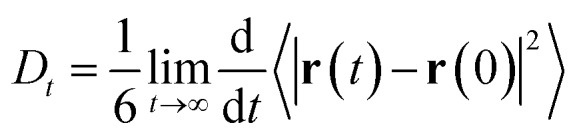
where ***r***(*t*) is the position vector of each atomic center at time *t*, and the angled brackets indicate an average over the NVE ensemble. We find that the predicted diffusion coefficients calculated using the last 10 ps of the MSD curves for revPBE-D3 and B97M-rV bracket the experimental value of 2.3 × 10^–9^ m^2^ s^–1^ (1.9 × 10^–9^ m^2^ s^–1^ and 2.9 × 10^–9^ m^2^ s^–1^, respectively) while M06-L-D3 shows a highly suppressed value of 0.3 × 10^–9^ m^2^ s^–1^. Although the energy is well conserved during the NVE runs, we did observe that the average temperature for the different functionals differed for each simulation: 306.3, 301.9, and 290.7 K for revPBE-D3, B97M-rV, and M06-L-D3, respectively. The lower average temperature of M06-L-D3 may be partly responsible for the lower diffusivity that we observe, although qualitatively the result will remain the same since a temperature drop of 10 K corresponds to a slowing of diffusivity by 10% at most.

It is well known that finite size effects on diffusivity are relevant for system sizes of the same order as the ones simulated here (64 water molecules), with diffusivity increasing by up to ∼15–30% when extrapolated to the infinite system size limit.^123^ Although we have not performed the finite size corrections, since it would require carrying out multiple extra simulations for each functional, it is clear that correcting for finite size effects would improve the diffusional results for revPBE-D3 and M06-L-D3, while it would worsen the agreement for B97M-rV.

**Fig. 5:**
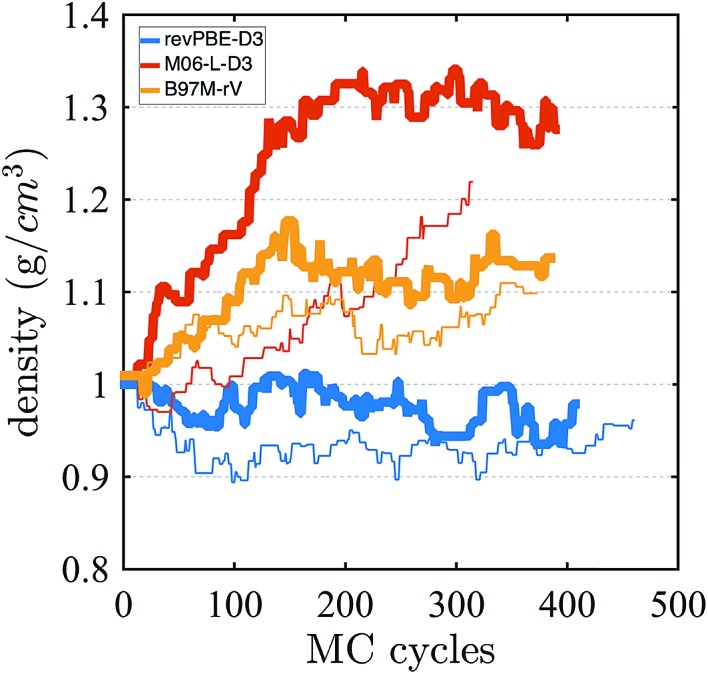
Density of water from AI-HMC simulations. The thick and thin lines correspond to simulations performed with energy cutoffs of 800 Ry and 400 Ry, respectively.

Finally, we investigate the equilibrium density at ambient pressure and temperature using AI-HMC since the computational implementation for AIMD-NPT is not available in CP2K for meta-GGAs. We investigate the density for two different energy cutoffs, 800 Ry and 400 Ry, the latter value corresponding to the cutoff used in the AIMD simulations in the isochoric ensembles. The evolution of the density as a function of the MC cycles is shown in Fig. 5. The density of M06-L-D3, for 800 Ry, converges around 1.30 g cm^–3^, which is much higher than the experimental value of 0.997 g cm^–3^. The 400 Ry case does not converge in the time of the simulation, suggesting that larger energy cutoffs may facilitate fast convergence. For revPBE-D3 and B97M-rV, reasonable converge is achieved for both energy cutoffs. The functional revPBE-D3, with a density of 0.97 g cm^–3^ at 800 Ry and 0.94 g cm^–3^ at 400 Ry, is in reasonable agreement with a previous study reporting a value of 0.95 g cm^–3^ with an older implementation of Grimme's dispersion corrections,^60^ but its density is lower than the experimental value. B97M-rV appears to converge at a higher value than experiment: ∼1.12 g cm^–3^ for 800 Ry, and ∼1.08 g cm^–3^ for 400 Ry. The trends in the densities are consistent with the RDF and IR spectrum that suggest that the water network of revPBE-D3 is slightly expanded due to stronger and more directional hydrogen bonds, whereas the weaker hydrogen bonds and higher interstitial populations in the RDF for the meta-GGAs increase the overall density, particularly in the case of M06-L-D3.

The glassy dynamics observed for M06-L-D3 at 1.0 g cm^–3^ might on the face of it seem at odds with its equilibrium density being much higher: ∼1.3 g cm^–3^. Since we did not calculate the diffusion constant at the equilibrium density, there are two interpretations in regards to this result. The first is that the slow diffusivity that we observe at the much lower density of 1 g cm^–3^ could still be much higher than that of an arrested phase at the equilibrium density. Alternatively, water is known to exhibit diffusion anomalies, *i.e.*, unlike a normal liquid, the self-diffusion of water actually increases about the ambient density value. Thus perhaps M06-L-D3 gets this qualitative behavior right, although in absolute terms the self-diffusion coefficient at 1.0 g cm^–3^ is in significant disagreement with experiment.

To further understand the influence of the intrinsic quality of the functionals on the liquid water state properties, we calculated the harmonic vibrational frequencies of the water dimer, trimer, tetramer, pentamer, and 4 hexamers, using all-electron simulations in Q-Chem, and compared the results to CCSD(T) benchmark calculations^124^,^125^ (Table S7†). It is worth mentioning that Howard *et al.*^124^ calculated the vibrational frequencies of water clusters using ∼60 functionals, and M06-L-D3 was found to be the best performing method from the semi-local functionals considered. Our results for M06-L-D3 agree with those reported in that paper.

In Table 2 we report the intrinsic functional errors on the bonded O–H vibrational frequencies (Δ*ω*_intrinsic_ = *ω*_CCSD(T)_ – *ω*_DFT_) as averages over each cluster size, from the dimer to the pentamer. Whereas B97M-rV is highly accurate, and M06-L-D3 performs reasonably well, revPBE-D3 has an average intrinsic red-shift error of ∼215 cm^–1^, consistent with the fact that water monomers of revPBE-D3 are too deformable (*i.e.* the bonds are too soft).^55^ The red shift observed in the liquid of revPBE-D3 relative to the meta-GGAs is therefore likely due to the intrinsic error of this GGA functional. The intrinsic error of the functionals in the vibrational frequencies is very similar when *ω*_DFT_ are calculated using the same conditions as for the AIMD simulations: GPW in CP2K, periodic simulation cell, the mTZV2P basis set, and a 400 Ry energy cutoff (Table S9†). As expected, the behavior of M06-L-D3 is erratic among the different water clusters at this low plane wave cutoff. This inconsistent behavior diminishes when we increase the energy cutoff to 800 Ry, confirming the sensitivity of M06-L-D3 to the quality of the grid.

**Table 2:** Analysis of the intrinsic errors of the density functionals with respect to CCSD(T) reference values, Δ*ω*_intrinsic_, and estimates of the shifts due to NQE, Δ*ω*_NQE_, in the bonded O–H vibrational frequencies of four different water clusters. The *ω*_DFT_ were calculated in Q-Chem with the def2-QZVPPD basis set and a (250 974) grid. Units are cm^–1^

Cluster bonded O–H errors	Δ*ω*_intrinsic_^*a*^ = *ω*_CCSD(T)_ – *ω*_DFT_			Δ*ω*_NQE_^*b*^ = *ω*_exp_ – *ω*_AIMD_ – Δ*ω*_intrinsic_		
	revPBE-D3	B97M-rV	M06-L-D3	revPBE-D3	B97M-rV	M06-L-D3
Dimer	168	–5	29	–303	–202	–226
Trimer	191	–4	46	–252	–196	–236
Tetramer	240	–16	51	–270	–236	–205
Pentamer	252	–17	51	–374	–249	–310

	〈ΔΔ*ω*_NQE_〉_clusters_^*b*^			Predicted bonded O–H stretch in liquid		
All clusters	–300	–220	–244	3104	3402	3331

^*a*^Negative values correspond to blue shifts.

^*b*^Negative values correspond to red shifts upon the treatment of nuclei dynamics quantum mechanically. The bonded OH frequencies have been averaged, and the values in the table are calculated using the raw data given in Tables S8 and S9.

Using the averaged experimental values^126^ (*ω*_ex_), the vibrational frequencies calculated from AIMD simulations that include anharmonic effects (*ω*_AIMD_), and the intrinsic functional error that we just calculated (Δ*ω*_intrinsic_), we can compute the shifts that the bonded O–H vibrational frequencies for each water cluster will experience upon simulation of NQE (Δ*ω*_NQE_ = *ω*_exp_ – *ω*_AIMD_ – Δ*ω*_intrinsic_). Comparison of the O–H vibrational frequencies between experiments and AIMD simulations confirms that the classically generated cluster frequencies are blue-shifted (Table S10†). Had the AIMD simulations of the water clusters been performed with nuclear quantum dynamics instead, we would expect the bonded O–H stretches peaks to red shift somewhere between ∼220–300 cm^–1^ depending on the functional (Table 2).

The intrinsic functional error and the estimations of frequency shifts expected with NQEs help interpret the rich IR spectrum in liquid water shown in Fig. 3, at least for the O–H stretch mode. While from classical AIMD we find blue shifts of ∼200–300 cm^–1^ for the O–H stretching modes relative to experiment for both meta-GGAs, the expected red-shift to lower frequencies due to NQEs would bring B97M-rV into excellent agreement with the experimental IR spectra for the liquid, and slightly over correct the M06-L-D3 functional by a ∼70 cm^–1^ (Table 2). By contrast, the functional revPBE-D3 exhibits the benefit of a fortuitous cancellation of its large intrinsic error that roughly matches the red shift due to NQEs, ignored by using classical trajectories. Thus, unlike the meta-GGAs, inclusion of quantum delocalization is expected to red shift the higher frequency modes of revPBE-D3 even further, thereby significantly diminishing agreement with the experimental IR spectra.

Interestingly, we observe in the liquid that the librational modes for all three functionals are red-shifted ∼80 cm^–1^ with respect to experiments, indicating that the intermolecular hydrogen-bonded network permits more permissive rocking motions. Since all three functionals have small intrinsic error in the librational region of the spectra for clusters, it might be expected that greater proton delocalization will strengthen the directional nature of the hydrogen-bonds to tighten the network, resulting in a blue-shift of the lower frequency modes that will improve the agreement with the experiment.

## Discussion

It has been previously recognized that computational models that accurately capture the potential energy and dipole moment surfaces of water, *e.g.* MB-Pol^25^ and WHBB,^24^ require NQE to complete the correct and accurate accounting of water properties.^127^ Our observations are consistent with this well known result, *i.e.* the interpretation that the meta-GGAs, in particular B97M-rV, offer a much more accurate description of the underlying PES of water than the GGA revPBE-D3, and hence the need for quantum nuclear dynamics to exploit the full potential of the better DFT functionals. This is a complementary statement to the fact that less accurate potential energy surfaces, such as that of revPBE-D3, will in fact be made worse with the addition of NQEs, as was originally shown by many groups in the failure of empirically-adjusted classical force fields to correctly reproduce the OH-stretch region when used in approximate quantum calculations of the IR spectrum.^128^–^130^

More specifically, the vibrational analysis of water clusters implies that the meta-GGA functionals investigated here should be blue-shifted in the liquid when trajectories are run with classical dynamics. In fact, this is what we observe in the IR spectrum of liquid water, indicating the meta-GGAs require the accompanying physics of NQE to complete them. The resulting red shifts in the monomer vibrational modes under quantum dynamics would thereby promote stronger intermolecular hydrogen bonding, which in turn would result in favorable changes in other liquid state properties.

*Ab initio* path integral simulations of liquid water will be ultimately needed to unequivocally validate the predictions presented here. Recent work by a number of research groups has broken the barrier to sampling that allows NQE to be simulated with an *ab initio* PES at a bracing but still acceptable cost,^101^,^131^ and has elucidated the qualitative changes to the RDF that are expected to arise when NQE is included. Typically, with the introduction of NQE, the rise in the first peak occurs at a slightly shorter distance with a corresponding lowering of the first O–O peak height. The minimum following the first peak of the RDF is deeper in the quantum case due to destabilization of interstitial configurations, which thereby enhances the second peak. The over-structuring in the *g*_OH_(*r*) is also corrected with NQE included. Thus, we anticipate that quantum dynamics would improve the structural results for the meta-GGA functionals by shifting the 1^st^ peak to smaller *r*, and destabilize the interstitial configurations in the trough region of *g*_OO_(*r*) in favor of increasing the density in the region at ∼4.5 Å that gives rise to the counterintuitive structure-enhancing effect of quantum delocalization.^121^,^131^ Even though the revPBE-D3 functional is in near quantitative agreement with experiment for the *g*_OO_(*r*) without NQE, we expect that it will worsen when NQEs are included.

In addition, stronger and more directional hydrogen bonding would expand the hydrogen-bonded network and lower the density of B97M-rV and M06-L-D3; correspondingly it would be expected that revPBE-D3 would move toward a density that more severely underestimates the experimental density, much like the usual GGA behavior exhibited by BLYP and PBE. It might also be expected that stronger hydrogen bonds would slow the diffusion in water by increasing the caging regime, thereby counteracting the increase in diffusion once finite size corrections are made.

## Conclusions

An accurate *ab initio* description of water in the condensed phase is essential to predict processes in regimes that are inaccessible or poorly described by empirical force fields, such as chemical reactions or nanoconfined environments. If we are to believe that the extensive benchmarking of density functionals and wavefunction models in the gas phase are relevant, and that nuclear quantum effects (NQEs) are important,^128^–^130^ then the only way forward is to continue to push the boundaries of what is possible toward better DFT functionals in the condensed phase at acceptable cost to enable sampling. Due to their high computational cost, most AIMD studies have been carried out at the GGA level of density functional theory. But despite notable advances over the past years—in particular the addition of dispersion corrections—capturing the physical properties of water using semi-local functionals remains challenging. Stimulated by the advent of less costly implementations of exact Hartree–Fock exchange for periodic systems, hybrid functionals have started to be used and show great promise;^132^ however, the associated computational cost is still too high to afford routine calculations.

The meta-GGA level of theory has been generally neglected in condensed phase simulations of water. The poor results of early studies^76^,^99^ that used the Tao–Perdew–Staroverov–Scuseria (TPSS) functional,^133^ together with the fact that meta-GGAs still suffer from delocalization error, have been sources of discouragement to extending their use in the condensed phase. However, some more recent meta-GGA functionals can offer similar accuracy to hybrids for non-covalent interactions at a fraction of the cost. Here, we have investigated the condensed phase properties of water using some of the most accurate meta-GGAs corrected for dispersion available, B97M-rV and M06-L-D3, and compared their performance with that of revPBE-D3 that serves as the current standard in classical AIMD simulations of water.

While the overall performance of the revPBE-D3 functional appears, on the face of it, to be better than the meta-GGAs, the work presented here has shown that this GGA is finely balanced between a set of fortuitous cancellation of errors in condensed phase simulations. While revPBE-D3 predicts the IR spectra of liquid water with surprising accuracy, we show directly using high-level benchmarks for water cluster data up through the pentamer that this is due to the intrinsic error of the functional, which is systematically red-shifted, and to neglecting quantum effects in the nuclear dynamics. By contrast, while the intrinsic errors of the meta-GGAs are 1–2 orders of magnitude smaller, we show that estimates of nuclear quantum effects—defined as the difference between the experimental mid-IR data and the classical simulations over the same water cluster set—would bring them, in particular B97M-rV, into likely excellent agreement with the IR spectra of the liquid, while significantly worsening the result for revPBE-D3 due to further red-shifting. Although M06-L-D3 is intrinsically a good functional, there are several hints from our benchmark calculations (*e.g.* Tables S5 and S9†) that it suffers from greater sensitivity to energy cutoffs (*i.e.* grid sensitivity), which is likely the reason for the poor performance in the AIMD simulations. By contrast, the B97M-rV functional is less sensitive to these numerical issues, and has a more coherent story as to how the inclusion of NQEs would move all water properties toward better agreement with experiment, including long time scale diffusivity.

But the question that remains is “how much” correction will be possible once NQEs are accounted for? A recent review^132^ has highlighted that the explicit representation of quantum delocalization has well-documented influence on structure, density, and long time-scale diffusivity of liquid water – that is a tradeoff of both the strengthening and weakening of hydrogen-bonds. We hope to report on a future study directed toward quantum dynamical simulations of the meta-GGAs to verify the predictions made here using water cluster data and their extensibility to bulk liquid water.

## Supplementary Material

Supplementary information
