# Supplementary material for: Ab initio molecular dynamics simulations of liquid water using high quality meta-GGA functionals
Source: Chem Sci. 2017 Feb 27;8(5):3554–65. doi: 10.1039/c6sc04711d (PMC6092720; doi:10.1039/c6sc04711d)
Supplement: Supplementary file 1 [file SC-008-C6SC04711D-s001.pdf]

Electronic Supplementary Material (ESI) for Chemical Science.
This journal is © The Royal Society of Chemistry 2017

Supplementary Information for Ab Initio Molecular Dynamics Simulations of Liquid Water using High Quality Meta-GGA Functionals

Luis Ruiz Pestana[1], Narbe Mardirossian[2], Martin Head-Gordon[2], Teresa Head-Gordon[1,3]

1. Validation of the meta-GGA implementation in the CP2K code.

Our original interest in examining the meta-GGA functionals were motivated by their very good performance in reproducing a wide range of high quality reference data[1, 2]. Therefore an important validation step is to ensure that implementation of these functionals through LIBXC[3] in the CP2K code, that uses the mixed Gaussian and plane waves (GPW) approach with pseudopotentials (PPs), is in sufficient agreement with the all-electron reference wavefunction calculation in the Q-Chem software package[4]. For this purpose, we analyze the errors in the binding energies by the different methodologies of the dimers in the S22[5] dataset (recently updated[6]) that contains 8 dispersion-bound, 7 hydrogen-bonded, and 7 mixed non-covalent interactions.

To assess the PP error, we compare the all-electron wavefunction method implemented in Q-Chem, the Gaussian augmented plane wave (GAPW) all-electron method[7] implemented in CP2K, and the Gaussian plane wave (GPW) pseudopotential method also implemented in CP2K, for the revPBE-D3 functional (Table S1). For the GPW calculations we employ Goedecker-Teter-Hutter (GTH) pseudopotentials[8, 9], in particular the GTH PP optimized for the functional PBE, PBE-PP. Because the D3 corrections that we apply to revPBE and M06-L are with zero-damping[10], in the following, we refer to those simply as D3, instead of the alternative D3(0) notation. The basis set (BS) aug-cc-pVTZ was used for the Q-Chem and GAPW calculations, and the aug-QZV3P for the GPW calculations. We determined a mean absolute error (MAE) of 0.048 kcal/mol between GAPW and Q-Chem, which rises to 0.136 kcal/mol for GPW. This error of ~$0.1k_BT$ is likely the best that any density functional can be represented by plane waves, and furthermore we believe is acceptable for our intended study in the bulk phase.

Next we compared the GPW performance of B97M-rV and M06-L-D3, against all electron Q-Chem benchmark calculations (Table S2). In these GPW calculations we use a plane

wave energy cutoff (E_{cut}) of 1200 Ry, an aug-QZV3P BS, and the PBE-PP for both meta-GGAs. The mean signed error (MSE) is very small, just 0.07 kcal/mol and -0.05 kcal/mol for M06-L-D3 and B97M-rV respectively. This result suggests, first, that the GPW representation of the meta-GGAs using large E_{cut} and large BS is very accurate, and second, that the error introduced by a PP that was not explicitly optimized for each specific functional is very small.

To further prove the latter point, we have compared the binding and relative energies of the complexes in the WATER20 dataset[11] for the functional M06-L-D3 using the PBE-PP and an optimized PP that we obtained following an optimization procedure available in CP2K (Table S3). We observe that the difference between both PPs in the relative energies is negligible. However, the optimized PP results in substantially larger errors in the binding energies. A closer look reveals a shift of the optimized PP of ~5 kcal/mol in the binding energies with respect to the PBE-PP. This shift is in the wrong direction, which ultimately translates in higher values of the MSE and RMSE. In all the simulations presented in the manuscript we have used the PBE-GTH-PP for all the functionals. Together, these benchmarks allow us to proceed with some confidence in examining the behavior of the functionals in the condensed phase.

2. Validation of the three functionals under conditions relevant for the condensed phase.

Although the meta-GGA functionals M06-L-D3 and B97M-rV were developed, optimized, and intended for refined DFT grids and large BS, the condensed phase simulations using the GPW approach are run under significantly different protocols, such as BS of moderate size and tractable E_{cut}. To gain further insight into the performance of the meta-GGAs and revPBE-D3 under such conditions, we calculate the errors in the binding energies of the S22[5] and WATER20[11] data sets, and compare them to the errors from Q-Chem calculations, where the def2-QZVPPD basis set is used without counterpoise corrections, along with a (99,590) grid for local exchange-correlation functionals and the SG-1 grid for the VV10 nonlocal correlation functional[12]. It is worth noting that we use updated reference values for both data sets[6, 13]. In the GPW calculations we use mDZVP or mTZV2P, and E_{cut}=400 Ry or E_{cut}=800 Ry, which are representative of our simulations in the NVT/NVE and NpT ensembles, respectively.

In Table S3 we evaluate the root mean-squared error (RMSE) and mean signed percentage error (MSPE) of the binding energies for the S22 dataset. According to our convention in the MSPE, positive values indicate over-binding with respect to the reference, while negative values indicate under-binding. The MSPE reflect a consistent trend expected from the basis set superposition

error (BSSE), i.e. any under-binding exhibited by the benchmark systematically improves with the smaller basis sets used in the GPW protocol, whereas any over-binding gets worse in the GPW implementation with typical condensed phase settings. In this regard, revPBE-D3 that has already a tendency to overbind degrades, whereas the MSPE in the meta-GGAs either substantially improve (M06-L-D3) or show minor shifts from small under-binding to small over-binding (B97M-rV). The overall errors do not appear to be sensitive to either BS or E_{cut}.

In the case of WATER20 shown in Table S4, the RMSE are considerably larger because the typical binding energies of the water clusters are an order of magnitude larger than those of the small complexes in the S22 dataset. In contrast to the S22 dataset, here both BS and E_{cut} play an important role, with larger BS and higher cutoffs leading to smaller errors, as expected. Interestingly, for mTZV2P and E_{cut}=800 Ry (the most accurate settings employed), the three functionals outperform the Q-Chem benchmarks, indicating that results from NpT simulations where we use those settings will not be strongly compromised by the condensed phase settings. By contrast, the mTZV2P and E_{cut}=400 Ry, which are the settings that we use to calculate properties such as radial distribution functions, vibrational spectra, and diffusion constant from AIMD simulations in the NVT or NVE ensembles, improve for revPBE-D3, but degrade significantly M06-L-D3, which appears to be very sensitive to E_{cut}.

Even so, binding energies may be a poor choice for validation for properties in the condensed phase, and so we also benchmark against the relative energies of isomerization for the WATER20 dataset in Table S5. Overall, the differences in errors between the Q-Chem and GPW are small. The grid sensitivity of M06-L-D3 is exposed again, where for example for mTZV2P, the RMSE increases from 2.78 to 4.18 (and the MSE from 0.84 to 1.74) going from 800 to 400 Ry.

3. Validation of the procedure for calculating the density in the NPT ensemble.

As discussed in the Methods section, constant pressure molecular dynamics simulations that rely on the calculation of the pressure virial have been reported to be particularly sensitive to BS and E_{cut}[14, 15]. We have studied the equilibrium density of water using revPBE-D3, the more affordable functional, for two different basis sets (mTZV2P and mDZVP) and in the mTZV2P case, for two different cutoffs: 1700 Ry and 800 Ry. Only the simulation with the highest cutoff and larger basis set (blue line in Figure S1) converges in the limited simulation time.

The density for revPBE-D3 with mTZV2P and 1700 Ry is 0.93 g/cm^3, in good agreement with previous studies (~0.95 g/cm^3). Although the pressure fluctuations are large (in the order of 7.5 kbar) due to the small system size, the average converges well to the applied external pressure of 1 atm (inset in Figure S1), in agreement with the virial theorem[16]. These pressure fluctuations are similar to those measured in other NpT simulations of ab-initio water performed using converged discrete variable representation basis sets and the functional BLYP[17]. Due to difficulties in the computational implementation, AIMD simulations in the NpT ensemble are not feasible for meta-GGAs in CP2K. For this reason, we use ab initio hybrid Monte Carlo (AI-HMC) as an alternative, possibly better method, to find the equilibrium density. We observe that the density of 0.97 g/cm^3 from AI-HMC (Figure 5) is in reasonable agreement with our AIMD-NpT simulation result of 0.93 g/cm^3, given the errors involved in the calculation. We have also calculated the density using the same BS and E_{cut} than for the isochoric simulations, and the results are also shown in Figure 5. We find that the equilibrium density shifts very slightly (~0.02 g/cm3) towards smaller values for all the functionals.

Table S1. *Binding energies and mean absolute errors (MAE) for the different implementations of the revPBE-D3 functional evaluated on the S22 dataset.* We compare results from all-electron Q-Chem calculation using an aug-cc-pVTZ basis set, all-electron (all-e) CP2K calculations using the aug-cc-pVTZ basis set, and pseudopotential (PP) CP2K calculations using the aug-QZV3P basis set. The units are kcal/mol.

S22 complex	Q-Chem	CP2K (all-e)	CP2K (PP)
**Hydrogen-bonded**			
NH_3 dimer	-3.40	-3.38	-3.42
H_2O dimer	-5.40	-5.40	-5.51
Formic acid dimer	-19.78	-19.75	-19.68
Formamide dimer	-16.47	-16.35	-16.33
Uracil dimer (planar)	-21.02	-20.75	-20.59
2-pyridone2-aminopyridine	-18.23	-18.15	-18.05
Adeninethymine WC	-17.54	-17.29	-17.13
**Dispersion-bound**			
CH_4 dimer	-0.83	-0.83	-0.80
C_2H_4 dimer	-1.69	-1.69	-1.65
Benzene-CH_4	-1.65	-1.65	-1.58
Benzene dimer (parallel)	-2.53	-2.53	-2.37
Pyrazine dimer	-3.72	-3.73	-3.57
Uracil dimer (stacked)	-9.33	-9.24	-9.02
Indolebenzene	-3.93	-3.92	-3.69
Adeninethymine	-10.20	-10.14	-9.84
**Mixed complexes**			
C_2H_4-C_2H_2	-1.96	-1.96	-1.94
Benzene-H_2O	-3.67	-3.67	-3.68
Benzene-NH_3	-2.61	-2.61	-2.58
Benzene-HCN	-4.64	-4.65	-4.62
Benzene dimer (t-shaped)	-2.80	-2.81	-2.72
Indolebenzene	-5.68	-5.71	-5.62
Phenol dimer	-6.94	-6.99	-6.90
**MAE wrt Q-Chem**		**0.048**	**0.136**
**MAE wrt CP2K all-e**			**0.101**

Table S2. *Binding energies and mean signed errors (MSE) of the S22 dataset for B97M-rV and M06-L-D3.* The MSE is calculated between CP2K results (1200 Ry, aug-QZV3P, GTH-PP) and all-electron Q-Chem calculations (def2-QZVPPD without counterpoise corrections). The units are kcal/mol.

**S22 complex**	**B97M-rV**		**M06-L-D3**	
	**Q-Chem**	**CP2K**	**Q-Chem**	**CP2K**
**Hydrogen-bonded**				
NH_3 dimer	-3.14	-3.14	-2.75	-2.76
H_2O dimer	-5.03	-5.03	-4.69	-4.79
Formic acid dimer	-18.79	-18.34	-19.08	-19.20
Formamide dimer	-15.74	-15.50	-15.83	-15.95
Uracil dimer (planar)	-20.26	-20.08	-19.67	-20.00
2-pyridone2-aminopyridine	-16.56	-16.46	-16.66	-16.92
Adeninethymine WC	-16.02	-15.94	-15.83	-16.11
**Dispersion-bound**				
CH_4 dimer	-0.46	-0.47	-0.55	-0.44
C_2H_4 dimer	-1.38	-1.42	-1.29	-1.20
Benzene-CH_4	-1.43	-1.47	-1.12	-1.00
Benzene dimer (parallel)	-2.80	-2.92	-2.75	-2.41
Pyrazine dimer	-4.14	-4.22	-3.82	-3.49
Uracil dimer (stacked)	-10.19	-10.42	-9.59	-9.17
Indolebenzene	-4.74	-4.98	-4.53	-4.12
Adeninethymine	-12.35	-12.69	-11.90	-11.44
**Mixed complexes**				
C_2H_4-C_2H_2	-1.55	-1.67	-1.07	-1.07
Benzene-H_2O	-3.21	-3.31	-2.97	-2.76
Benzene-NH_3	-2.25	-2.31	-2.01	-1.84
Benzene-HCN	-4.33	-4.55	-3.87	-3.84
Benzene dimer (t-shaped)	-2.49	-2.70	-2.18	-2.09
Indolebenzene	-5.24	-5.57	-4.77	-4.76
Phenol dimer	-6.75	-6.84	-6.44	-6.40
**MSE wrt Q-Chem**		**-0.054**		**0.073**

Table S3. *Root mean-squared error (RMSE) and mean signed error (MSE) of the binding and relative energies of the WATER20 dataset by the functional M06-L-D3, used with two different GTH pseudopotentials: PBE-PP and a functional-optimized PP.* The units are kcal/mol.

		**Binding energies**				**Relative energies**			
		**PBE-PP**		**Optimized PP**		**PBE-PP**		**Optimized PP**	
**BS**	**E_{cut} (Ry)**	**RMSE**	**MSE**	**RMSE**	**MSE**	**RMSE**	**MSE**	**RMSE**	**MSE**
mDZVP	400	16.27	-15.64	21.61	-21.16	4.98	2.14	4.91	2.27
mDZVP	800	4.56	-2.88	8.83	-8.15	3.72	1.18	3.62	1.31
mTZV2P	400	11.58	-10.94	17.11	-16.70	4.18	1.74	4.15	1.86
mTZV2P	800	3.32	1.99	4.31	-3.48	2.78	0.84	2.72	0.97

Table S4. *Root mean-squared error (RMSE) and mean signed percentage error (MSPE) of the S22 dataset binding energies.* The mTZV2P and 400 Ry red-shaded row corresponds to the settings that were used for the AIMD simulations in the NVT and NVE ensembles. The mTZV2P and 800 Ry blue-shaded row corresponds to the settings employed in the AI-HMC simulations. The units are kcal/mol.

		**revPBE-D3**		**B97M-rV**		**M06-L-D3**	
**BS**	**E_{cut} (Ry)**	**RMSE**	**MSPE**	**RMSE**	**MSPE**	**RMSE**	**MSPE**
mDZVP	400	0.49	4.84%	0.43	2.80%	0.54	0.51%
mDZVP	800	0.47	5.51%	0.44	3.21%	0.56	1.53%
mTZV2P	400	0.52	5.48%	0.44	1.34%	0.54	1.65%
mTZV2P	800	0.49	6.15%	0.44	1.72%	0.55	2.62%
**Q-Chem**		0.61	2.50%	0.29	-1.72%	0.47	-7.96%

Table S5. *Root mean-squared error (RMSE) and mean signed percentage error (MSPE) of the WATER20 dataset binding energies.* The mTZV2P and 400 Ry red-shaded row corresponds to the settings that were used for the AIMD simulations in the NVT and NVE ensembles. The mTZV2P and 800 Ry blue-shaded row corresponds to the settings employed in the AI-HMC simulations. The units are kcal/mol.

		**revPBE-D3**		**B97M-rV**		**M06-L-D3**	
BS	E_{cut} (Ry)	RMSE	MSPE	RMSE	MSPE	RMSE	MSPE
mDZVP	400	3.80	1.68%	6.25	3.00%	16.27	7.56%
mDZVP	800	4.22	1.96%	4.81	2.28%	4.56	1.36%
mTZV2P	400	3.03	-1.12%	2.80	1.30%	11.58	5.29%
mTZV2P	800	2.34	-0.83%	1.53	0.65%	3.32	-0.99%
**Q-Chem**		9.70	-4.66%	1.64	-0.74%	4.15	-1.44%

Table S6. *Root mean-squared error (RMSE) and mean signed error (MSE) of the WATER20 dataset relative energies.* The mTZV2P and 400 Ry red-shaded row corresponds to the settings that were used for the AIMD simulations in the NVT and NVE ensembles. The mTZV2P and 800 Ry blue-shaded row corresponds to the settings employed in the AI-HMC simulations. The units are kcal/mol.

		**revPBE-D3**		**B97M-rV**		**M06-L-D3**	
BS	E_{cut} (Ry)	RMSE	MSE	RMSE	MSE	RMSE	MSE
mDZVP	400	1.94	-1.02	1.07	-0.57	4.98	2.14
mDZVP	800	1.48	-0.72	0.99	-0.28	3.72	1.18
mTZV2P	400	2.22	-1.12	1.13	-0.75	4.18	1.74
mTZV2P	800	1.77	-0.82	0.87	-0.47	2.78	0.84
**Q-Chem**		1.31	-0.56	0.64	-0.16	3.01	0.52

Table S7. *Vibrational frequencies of water clusters from the dimer (n = 2) to the hexamer (n = 6) calculated in Q-Chem with the def2-QZVPPD basis set and a (250,974) grid.* The modes are separated in hydrogen bonded-OH stretch (BS), free-OH stretch (FS), angle bend (AB), and librational modes (LM), which include all the modes with frequencies < 1000 cm^{-1}. The units are cm^{-1}. For the eight water clusters, full geometry optimizations were carried out with each functional, followed by a harmonic frequency calculation (using 6N¬atoms analytic gradient calculations to compute the Hessian via finite difference). The geometry optimizations were carried out until the maximum gradient component was smaller than $50x10^{-6}$, and either the maximum atomic displacement was smaller than $50x10^{-6}$ a.u. or the absolute energy change was smaller than $50x10^{-8}$ Eh. The def2-QZVPPD basis set (132 basis functions per water molecule) was used for the frequency calculations, along with a (250,974) integration grid (250 radial shells with 974 grid points per shell) for local exchange-correlation functionals, and the SG-1 integration grid for the VV10 nonlocal correlation functional. The basis set and integration grid were chosen after carrying out extensive tests on the water dimer using eight different basis sets (aug-cc-pVDZ, aug-cc-pVTZ, aug-cc-pVQZ, aug-pc-1, aug-pc-2, aug-pc-3, pc-3, def2-QZVPPD) and four different integration grids ((75,302), (99,590), (250,974), (500,1454)) with all three functionals. The def2-QZVPPD/(250,974) combination was chosen as it was the most economical choice that resulted in a maximum absolute error of 5 cm^{-1} with respect to aug-pc-3/(500,1454) for the 12 vibrational modes of the water dimer, for all three functionals.

n	**Name**	**Mode**	**Ref.[18]**	**B97M-rV**	**M06-L-D3**	**revPBE-D3**
2	dimer	LM	126	126	137	119
2	dimer	LM	143	142	151	150
2	dimer	LM	149	151	163	157
2	dimer	LM	185	186	198	171
2	dimer	LM	348	353	369	365
2	dimer	LM	614	602	628	592
2	dimer	AB	1651	1687	1671	1604
2	dimer	AB	1671	1703	1689	1620
2	dimer	BS	3751	3757	3723	3584
2	dimer	FS	3827	3846	3841	3695
2	dimer	FS	3913	3930	3933	3774
2	dimer	FS	3933	3950	3955	3796
3	trimer	LM	167	173	186	156
3	trimer	LM	179	179	189	164
3	trimer	LM	188	191	204	185
3	trimer	LM	196	200	210	201
3	trimer	LM	220	218	231	210
3	trimer	LM	234	243	250	246
3	trimer	LM	337	332	344	339
3	trimer	LM	349	344	358	356
3	trimer	LM	437	433	448	434
3	trimer	LM	561	554	576	553
3	trimer	LM	655	638	659	626
3	trimer	LM	844	846	886	862
3	trimer	AB	1660	1691	1676	1612

3	trimer	AB	1663	1693	1679	1616
3	trimer	AB	1685	1712	1698	1628
3	trimer	BS	3627	3633	3574	3430
3	trimer	BS	3683	3686	3639	3492
3	trimer	BS	3691	3693	3648	3506
3	trimer	FS	3904	3921	3921	3766
3	trimer	FS	3908	3925	3925	3769
3	trimer	FS	3908	3926	3926	3771
4	tetramer	LM	48	46	41	48
4	tetramer	LM	77	75	70	85
4	tetramer	LM	205	203	207	189
4	tetramer	LM	208	204	220	231
4	tetramer	LM	235	232	239	232
4	tetramer	LM	235	232	239	232
4	tetramer	LM	250	245	258	247
4	tetramer	LM	250	245	258	268
4	tetramer	LM	255	246	263	268
4	tetramer	LM	283	280	282	307
4	tetramer	LM	395	382	393	405
4	tetramer	LM	424	411	431	437
4	tetramer	LM	439	426	441	454
4	tetramer	LM	439	426	441	454
4	tetramer	LM	733	704	719	728
4	tetramer	LM	799	771	801	818
4	tetramer	LM	799	771	801	818
4	tetramer	LM	962	937	975	988
4	tetramer	AB	1666	1692	1676	1612
4	tetramer	AB	1678	1704	1689	1627
4	tetramer	AB	1678	1704	1689	1627
4	tetramer	AB	1704	1728	1715	1653
4	tetramer	BS	3472	3494	3415	3214
4	tetramer	BS	3557	3572	3507	3320
4	tetramer	BS	3557	3572	3507	3320
4	tetramer	BS	3591	3604	3543	3363
4	tetramer	FS	3899	3919	3920	3765
4	tetramer	FS	3899	3920	3921	3766
4	tetramer	FS	3899	3920	3921	3766
4	tetramer	FS	3900	3920	3922	3767
5	pentamer	LM	18	21	22	22
5	pentamer	LM	40	39	36	39
5	pentamer	LM	61	61	57	69
5	pentamer	LM	64	64	59	72
5	pentamer	LM	178	175	187	166
5	pentamer	LM	179	184	190	215
5	pentamer	LM	190	192	209	222
5	pentamer	LM	218	220	232	227

5	pentamer	LM	231	226	240	230
5	pentamer	LM	235	229	245	252
5	pentamer	LM	255	255	265	284
5	pentamer	LM	284	281	294	289
5	pentamer	LM	293	284	300	291
5	pentamer	LM	296	287	303	316
5	pentamer	LM	400	387	404	411
5	pentamer	LM	417	404	420	431
5	pentamer	LM	438	426	444	457
5	pentamer	LM	452	438	459	468
5	pentamer	LM	509	494	512	520
5	pentamer	LM	695	667	692	707
5	pentamer	LM	764	735	755	775
5	pentamer	LM	833	807	840	865
5	pentamer	LM	852	824	855	878
5	pentamer	LM	950	925	959	982
5	pentamer	AB	1669	1694	1679	1612
5	pentamer	AB	1678	1703	1689	1624
5	pentamer	AB	1688	1712	1698	1633
5	pentamer	AB	1704	1729	1717	1654
5	pentamer	AB	1711	1735	1723	1662
5	pentamer	BS	3441	3464	3385	3165
5	pentamer	BS	3514	3531	3464	3260
5	pentamer	BS	3522	3539	3471	3269
5	pentamer	BS	3562	3576	3515	3322
5	pentamer	BS	3569	3583	3521	3331
5	pentamer	FS	3898	3918	3921	3765
5	pentamer	FS	3900	3920	3922	3767
5	pentamer	FS	3902	3921	3924	3768
5	pentamer	FS	3903	3922	3925	3769
5	pentamer	FS	3904	3924	3926	3771
6	ring	LM	28	27	26	25
6	ring	LM	28	27	26	25
6	ring	LM	45	45	46	46
6	ring	LM	45	45	46	50
6	ring	LM	50	49	47	50
6	ring	LM	82	82	85	92
6	ring	LM	156	153	163	145
6	ring	LM	172	173	174	195
6	ring	LM	195	198	201	206
6	ring	LM	195	198	201	206
6	ring	LM	211	205	218	222
6	ring	LM	211	205	218	222
6	ring	LM	254	255	262	282
6	ring	LM	254	255	262	282
6	ring	LM	282	283	293	288

6	ring	LM	292	283	300	288
6	ring	LM	292	284	300	314
6	ring	LM	323	313	333	320
6	ring	LM	407	392	406	415
6	ring	LM	426	412	429	440
6	ring	LM	426	412	429	440
6	ring	LM	441	428	445	457
6	ring	LM	450	438	456	469
6	ring	LM	450	438	456	469
6	ring	LM	757	727	753	763
6	ring	LM	776	746	775	789
6	ring	LM	776	746	775	789
6	ring	LM	867	840	875	895
6	ring	LM	867	840	875	895
6	ring	LM	941	916	952	973
6	ring	AB	1665	1690	1673	1606
6	ring	AB	1676	1700	1685	1619
6	ring	AB	1676	1700	1685	1619
6	ring	AB	1701	1726	1714	1651
6	ring	AB	1701	1726	1714	1651
6	ring	AB	1716	1742	1731	1669
6	ring	BS	3440	3462	3384	3161
6	ring	BS	3505	3522	3455	3246
6	ring	BS	3505	3522	3455	3246
6	ring	BS	3554	3569	3508	3311
6	ring	BS	3554	3569	3508	3311
6	ring	BS	3570	3584	3525	3332
6	ring	FS	3901	3921	3923	3768
6	ring	FS	3901	3921	3924	3768
6	ring	FS	3901	3921	3924	3768
6	ring	FS	3901	3922	3924	3769
6	ring	FS	3901	3922	3924	3769
6	ring	FS	3901	3922	3924	3769
6	book	LM	27	25	24	26
6	book	LM	37	37	38	40
6	book	LM	53	52	52	54
6	book	LM	67	65	62	70
6	book	LM	85	82	80	91
6	book	LM	156	158	170	131
6	book	LM	179	178	190	167
6	book	LM	189	188	198	187
6	book	LM	195	195	207	212
6	book	LM	225	221	238	225
6	book	LM	233	231	249	242
6	book	LM	245	243	259	258
6	book	LM	250	247	262	269

6	book	LM	271	267	268	280
6	book	LM	282	277	297	282
6	book	LM	291	281	298	292
6	book	LM	302	297	314	323
6	book	LM	377	363	379	383
6	book	LM	393	382	402	404
6	book	LM	432	416	432	438
6	book	LM	443	429	447	457
6	book	LM	467	455	474	485
6	book	LM	533	513	535	526
6	book	LM	601	585	603	601
6	book	LM	708	683	705	717
6	book	LM	735	715	737	753
6	book	LM	811	788	818	834
6	book	LM	829	806	832	853
6	book	LM	874	845	875	898
6	book	LM	989	965	1001	1020
6	book	AB	1661	1687	1670	1606
6	book	AB	1673	1698	1684	1619
6	book	AB	1675	1701	1686	1624
6	book	AB	1691	1716	1702	1640
6	book	AB	1702	1725	1711	1649
6	book	AB	1730	1751	1738	1674
6	book	BS	3386	3407	3323	3089
6	book	BS	3455	3472	3402	3186
6	book	BS	3503	3516	3449	3232
6	book	BS	3587	3601	3538	3356
6	book	BS	3637	3652	3598	3424
6	book	BS	3651	3662	3611	3439
6	book	FS	3768	3780	3748	3605
6	book	FS	3893	3916	3920	3766
6	book	FS	3898	3919	3921	3766
6	book	FS	3900	3920	3921	3766
6	book	FS	3900	3921	3922	3771
6	book	FS	3903	3923	3924	3773
6	cage	LM	42	39	33	42
6	cage	LM	56	53	47	57
6	cage	LM	74	72	67	74
6	cage	LM	100	96	93	95
6	cage	LM	127	131	145	99
6	cage	LM	153	157	172	126
6	cage	LM	185	187	203	163
6	cage	LM	194	197	215	190
6	cage	LM	210	206	219	211
6	cage	LM	223	218	224	213
6	cage	LM	231	227	233	215

6	cage	LM	234	228	244	233
6	cage	LM	242	235	251	252
6	cage	LM	253	255	261	272
6	cage	LM	281	271	290	286
6	cage	LM	293	288	291	295
6	cage	LM	383	365	373	368
6	cage	LM	395	381	393	397
6	cage	LM	437	422	437	422
6	cage	LM	453	437	454	447
6	cage	LM	467	447	461	461
6	cage	LM	534	517	542	530
6	cage	LM	553	536	558	564
6	cage	LM	620	606	628	616
6	cage	LM	682	665	689	685
6	cage	LM	717	694	716	716
6	cage	LM	774	753	780	797
6	cage	LM	790	767	794	802
6	cage	LM	852	832	864	867
6	cage	LM	975	946	978	998
6	cage	AB	1666	1691	1673	1611
6	cage	AB	1673	1700	1685	1627
6	cage	AB	1684	1706	1690	1630
6	cage	AB	1698	1721	1707	1645
6	cage	AB	1707	1732	1716	1655
6	cage	AB	1722	1745	1733	1669
6	cage	BS	3324	3346	3264	3037
6	cage	BS	3517	3530	3456	3272
6	cage	BS	3556	3573	3512	3334
6	cage	BS	3604	3619	3573	3368
6	cage	BS	3650	3664	3612	3424
6	cage	BS	3718	3733	3700	3561
6	cage	FS	3757	3770	3735	3593
6	cage	FS	3792	3801	3769	3639
6	cage	FS	3895	3916	3913	3763
6	cage	FS	3896	3918	3921	3771
6	cage	FS	3899	3920	3922	3771
6	cage	FS	3908	3927	3927	3776
6	prism	LM	61	61	58	59
6	prism	LM	70	67	63	64
6	prism	LM	74	71	69	74
6	prism	LM	98	110	125	81
6	prism	LM	112	123	138	93
6	prism	LM	149	151	165	126
6	prism	LM	173	174	188	156
6	prism	LM	178	178	194	159
6	prism	LM	212	210	209	192

6	prism	LM	217	213	230	208
6	prism	LM	238	232	248	221
6	prism	LM	246	241	257	227
6	prism	LM	275	269	272	251
6	prism	LM	284	274	283	266
6	prism	LM	287	281	291	279
6	prism	LM	357	348	360	350
6	prism	LM	367	355	369	361
6	prism	LM	420	405	416	415
6	prism	LM	427	413	429	416
6	prism	LM	462	448	463	451
6	prism	LM	491	480	493	472
6	prism	LM	530	514	529	524
6	prism	LM	547	535	553	544
6	prism	LM	612	596	610	613
6	prism	LM	638	622	644	630
6	prism	LM	675	657	673	683
6	prism	LM	711	687	708	723
6	prism	LM	823	796	821	821
6	prism	LM	868	844	870	859
6	prism	LM	1001	964	992	1013
6	prism	AB	1663	1687	1670	1610
6	prism	AB	1674	1697	1681	1621
6	prism	AB	1683	1702	1685	1625
6	prism	AB	1699	1724	1706	1647
6	prism	AB	1716	1742	1730	1664
6	prism	AB	1733	1755	1744	1677
6	prism	BS	3301	3334	3257	3016
6	prism	BS	3509	3521	3456	3308
6	prism	BS	3601	3625	3569	3376
6	prism	BS	3620	3638	3585	3422
6	prism	BS	3717	3735	3701	3554
6	prism	BS	3735	3753	3723	3564
6	prism	FS	3784	3794	3769	3631
6	prism	FS	3799	3810	3784	3650
6	prism	FS	3821	3826	3805	3693
6	prism	FS	3898	3918	3921	3773
6	prism	FS	3899	3918	3923	3774
6	prism	FS	3901	3920	3924	3778

Table S9. *Analysis of the intrinsic errors of the density functionals with respect to CCSD(T) reference values, $\Delta\omega_{intrinsic}$, and estimates of the shifts due to NQE, $\Delta\omega_{NQE}$, in the bonded O-H vibrational frequencies of four different water clusters.* The ω_{DFT} were calculated by a harmonic analysis in CP2K using a 25Å side cubic periodic simulation cell, the mTZV2P basis set, and an energy cutoff of 400 Ry. For M06-L-D3, we also do the calculation using a cutoff of E_{cut} = 800 Ry (light blue shaded column). Units are cm^{-1}.

Cluster Bonded O-H Errors	revPBE-D3	B97M-rV	M06-L-D3		revPBE-D3	B97M-rV	M06-L-D3	
	$\Delta\omega_{intrinsic}$[a] $= \omega_{CCSD(T)} - \omega_{DFT}$				$\Delta\omega_{NQE}$[b] $= \omega_{exp} - \omega_{AIMD} - \Delta\omega_{intrinsic}$			
**Dimer**	195	-14	153	8	-330	-193	-350	-205
**Trimer**	221	-18	-112	70	-282	-182	-78	-260
**Tetramer**	176	42	112	64	-206	-294	-266	-218
**Pentamer**	272	18	103	49	-394	-284	-362	-248
	$<\Delta\omega_{NQE}>_{clusters}$[b]				**Predicted bonded O-H stretch in liquid**			
**All Clusters**	-303	-238	-264	-248	3104	3402	3331	

[a] Negative values correspond to blue shifts

[b] Negative values correspond to red shifts upon the treatment of nuclei quantum mechanically. The bonded OH frequencies have been averaged, and the values in the table are calculated using the raw data given in Table S9.

Table S10. *OH stretch vibrational frequencies of water clusters from the dimer (n = 2) to the pentamer (n = 5) calculated from AIMD and compared to experiment.* The mTZV2P basis and E_{cut}=400 Ry were used. The modes are separated into bonded O-H stretch (BS) and free-OH stretch (FS). The units are cm^{-1}.

n	**MODE**	**Expt.** [19]	**B97M-rV**	**M06-L-D3**	**revPBE-D3**
2	FS	3735	3889	3948	3871
2	BS	3601	3808	3798	3736
3	FS	3726	3886	3919	3783
3	BS	3533	3733	3723	3594
4	FS	3714	3886	3922	3769
4	BS	3416	3668	3570	3446
5	FS	3714	3876	3922	3759
5	BS	3360	3626	3619	3482

Figure S1. *AIMD simulations in the NpT ensemble using revPBE-D3.* (a) Density as a function of time. (b) Probability distributions of the instantaneous pressure from the NpT simulations.

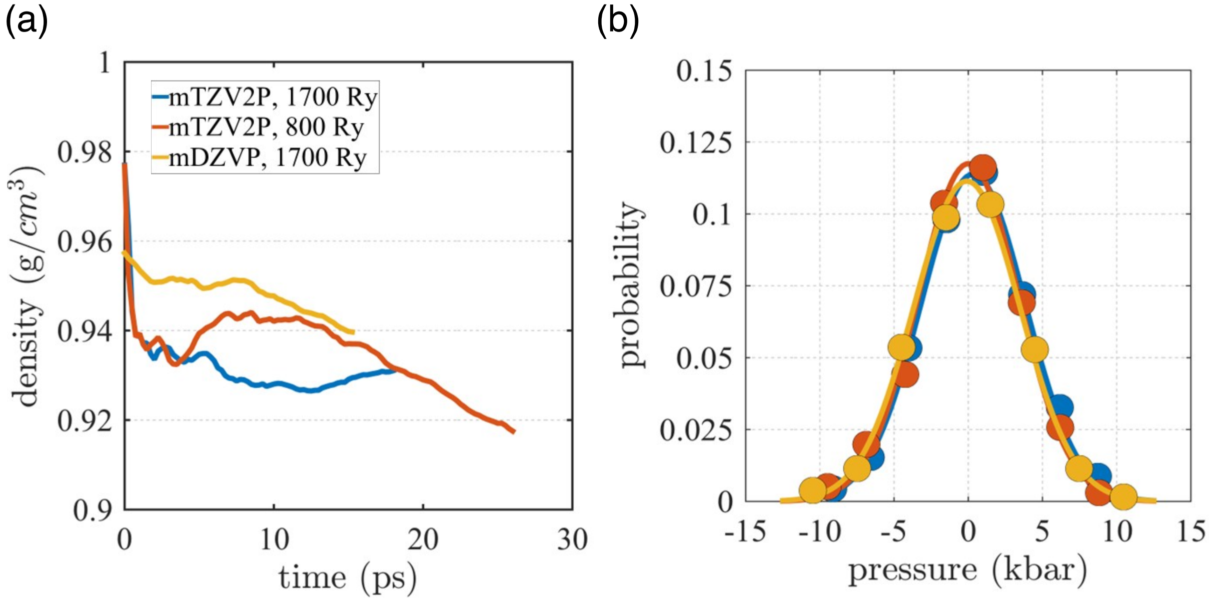

REFERENCES

1. N. Mardirossian and M. Head-Gordon, *The Journal of Chemical Physics*, 2015, **142**, 074111.

2. Y. Zhao and D. G. Truhlar, *The Journal of Chemical Physics*, 2006, **125**, 194101.

3. M. A. L. Marques, M. J. T. Oliveira and T. Burnus, *Computer Physics Communications*, 2012, **183**, 2272-2281.

4. Y. Shao, Z. Gan, E. Epifanovsky, A. T. B. Gilbert, M. Wormit, J. Kussmann, A. W. Lange, A. Behn, J. Deng, X. Feng, D. Ghosh, M. Goldey, P. R. Horn, L. D. Jacobson, I. Kaliman, R. Z. Khaliullin, T. Kuś, A. Landau, J. Liu, E. I. Proynov, Y. M. Rhee, R. M. Richard, M. A. Rohrdanz, R. P. Steele, E. J. Sundstrom, H. L. Woodcock, P. M. Zimmerman, D. Zuev, B. Albrecht, E. Alguire, B. Austin, G. J. O. Beran, Y. A. Bernard, E. Berquist, K. Brandhorst, K. B. Bravaya, S. T. Brown, D. Casanova, C.-M. Chang, Y. Chen, S. H. Chien, K. D. Closser, D. L. Crittenden, M. Diedenhofen, R. A. DiStasio, H. Do, A. D. Dutoi, R. G. Edgar, S. Fatehi, L. Fusti-Molnar, A. Ghysels, A. Golubeva-Zadorozhnaya, J. Gomes, M. W. D. Hanson-Heine, P. H. P. Harbach, A. W. Hauser, E. G. Hohenstein, Z. C. Holden, T.-C. Jagau, H. Ji, B. Kaduk, K. Khistyaev, J. Kim, J. Kim, R. A. King, P. Klunzinger, D. Kosenkov, T. Kowalczyk, C. M. Krauter, K. U. Lao, A. D. Laurent, K. V. Lawler, S. V. Levchenko, C. Y. Lin, F. Liu, E. Livshits, R. C. Lochan, A. Luenser, P. Manohar, S. F. Manzer, S.-P. Mao, N. Mardirossian, A. V. Marenich, S. A. Maurer, N. J. Mayhall, E. Neuscamman, C. M. Oana, R. Olivares-Amaya, D. P. O'Neill, J. A. Parkhill, T. M. Perrine, R. Peverati, A. Prociuk, D. R. Rehn, E. Rosta, N. J. Russ, S. M. Sharada, S. Sharma, D. W. Small, A. Sodt, T. Stein, D. Stück, Y.-C. Su, A. J. W. Thom, T. Tsuchimochi, V. Vanovschi, L. Vogt, O. Vydrov, T. Wang, M. A. Watson, J. Wenzel, A. White, C. F. Williams, J. Yang, S. Yeganeh, S. R. Yost, Z.-Q. You, I. Y. Zhang, X. Zhang, Y. Zhao, B. R. Brooks, G. K. L. Chan, D. M. Chipman, C. J. Cramer, W. A. Goddard, M. S. Gordon, W. J. Hehre, A. Klamt, H. F. Schaefer, M. W. Schmidt, C. D. Sherrill, D. G. Truhlar, A. Warshel, X. Xu, A. Aspuru-Guzik, R. Baer, A. T. Bell, N. A. Besley, J.-D. Chai, A. Dreuw, B. D. Dunietz, T. R. Furlani, S. R. Gwaltney, C.-P. Hsu, Y. Jung, J. Kong, D. S. Lambrecht, W. Liang, C. Ochsenfeld, V. A. Rassolov, L. V. Slipchenko, J. E. Subotnik, T. Van Voorhis, J. M. Herbert, A. I. Krylov, P. M. W. Gill and M. Head-Gordon, *Molecular Physics*, 2015, **113**, 184-215.

5. P. Jurecka, J. Sponer, J. Cerny and P. Hobza, *Physical Chemistry Chemical Physics*, 2006, **8**, 1985-1993.

6. M. S. Marshall, L. A. Burns and C. D. Sherrill, *The Journal of Chemical Physics*, 2011, **135**, 194102.

7. M. Krack and M. Parrinello, *Physical Chemistry Chemical Physics*, 2000, **2**, 2105-2112.

8. S. Goedecker, M. Teter and J. Hutter, *Physical Review B*, 1996, **54**, 1703-1710.

9. C. Hartwigsen, S. Goedecker and J. Hutter, *Physical Review B*, 1998, **58**, 3641-3662.

10. S. Grimme, J. Antony, S. Ehrlich and H. Krieg, *The Journal of Chemical Physics*, 2010, **132**, 154104.

11. G. S. Fanourgakis, E. Aprà and S. S. Xantheas, *The Journal of Chemical Physics*, 2004, **121**, 2655-2663.

12. O. A. Vydrov and T. Van Voorhis, *The Journal of Chemical Physics*, 2010, **133**, 244103.

13. T. Anacker and J. Friedrich, *Journal of Computational Chemistry*, 2014, **35**, 634-643.

14. M. J. McGrath, J. I. Siepmann, I. F. W. Kuo, C. J. Mundy, J. VandeVondele, J. Hutter, F. Mohamed and M. Krack, *ChemPhysChem*, 2005, **6**, 1894-1901.

15. M. J. McGrath, J. I. Siepmann, I. F. W. Kuo and C. J. Mundy, *Molecular Physics*, 2006, **104**, 3619-3626.

16. G. J. Martyna, D. J. Tobias and M. L. Klein, *The Journal of Chemical Physics*, 1994, **101**, 4177-4189.

17. Z. Ma, Y. Zhang and M. E. Tuckerman, *The Journal of Chemical Physics*, 2012, **137**, 044506.

18. J. C. Howard and G. S. Tschumper, *Journal of Chemical Theory and Computation*, 2015, **11**, 2126-2136.

19. F. Huisken, M. Kaloudis and A. Kulcke, *The Journal of Chemical Physics*, 1996, **104**, 17-25.
